# Chromium and cobalt intoxication mimicking mitochondriopathy

**DOI:** 10.1186/s42466-021-00141-0

**Published:** 2021-07-08

**Authors:** Tim W. Rattay, Torsten Kluba, Ludger Schöls

**Affiliations:** 1 Department for Neurodegenerative Diseases and Hertie-Institute for Clinical Brain Research, Center for Neurology, Hoppe-Seyler-Straße 3, 72076 Tübingen, Germany; 2 German Center of Neurodegenerative Diseases (DZNE), Tübingen, Germany; 3 Orthopedic Department, Klinikum Dresden, Dresden, Germany

**Keywords:** Chromium, Cobalt, Intoxication, Mitochondriopathy

## Abstract

A 53-year old male with a history of progressive visual impairment, hearing loss, peripheral neuropathy, poorly controlled diabetes mellitus, cardiomyopathy, and weight loss was referred to the rare disease center due to the suspicion of mitochondrial cytopathy. In line with mitochondrial dysfunction, lactate in CSF was increased. Genetic testing by whole-exome sequencing and mitochondrial DNA did not reveal a likely cause. The case remained unsolved until he developed pain in his right hip, where he had received total hip arthroplasty 12 years earlier. An orthopedic evaluation revealed substantial shrinkage of the head of the hip prosthesis. Due to metal-on-metal wear, debris chromium and cobalt levels in serum were massively increased and significantly improved with multisystemic impairment after exchanging the defective implant.

## Introduction

Mitochondrial disorders are regarded as medical chameleons and multisystem affection with CNS involvement including visual loss due to optic atrophy or retinopathy, external ophthalmoplegia, deafness, cerebellar dysfunction, neuromuscular manifestations such as peripheral neuropathy or myopathy, and extra-neural manifestations like diabetes mellitus, and cardiomyopathy (reviewed in [1]). However, working in a highly specialized outpatient clinic for rare neurogenetic diseases may mislead diagnostic procedures because these experts are oblivious to non-genetic disease causes.

## Case report

A 53-year old male was referred to our outpatient clinic for rare neurological disease with a six-month history of hyp- and dysesthesias pronounced in the lower extremities, weakness of legs, and progressive unsteadiness of gait with frequent near falls. Additional complaints included increased hair loss, reduced hair growth, and an undesired weight loss of 30 kg within the last year. His previous medical conditions included polycythemia (tested negative for the *JAK2*-mutation V617V), a two-year history of diabetes mellitus (HbA1c 12%) with several recent episodes of hyperglycemia (blood glucose > 300 mg/dl), a novel diagnosis (last 3 months) of non-obstructive cardiomyopathy (biopsy revealed moderately active chronic lymphocytic myocarditis), bilateral hearing loss (recognized beginning at age 45), and visual impairment which was progressive for at least 7 years. He underwent hip replacement 12-years ago due to severe hip arthrosis and had a revision after ceramic-head breakage. Family history was unremarkable for neurological or psychiatric diseases.

## Clinical, electrophysiological, and laboratory findings

His clinical examination revealed a decrease in visual acuity to 10% bilaterally (cc), bilateral eye abduction deficits (without diplopia) and vertical saccade slowing, saccadic pursuit movements, and a delayed direct and indirect pupillary response to light. There was weakness of proximal leg muscles (hip flexion 4/5 MRC, knee flexion 3–4/5) and wasting of the lower legs but preserved strength of foot extensors and flexors. Tendon reflexes were absent. Vibration sensation was reduced at ankles (2/8) and unaffected at hips and thumbs. Touch sensation was progressively reduced over the last 12 months (as reported by the patient); other sensory qualities were intact. With hands outstretched polyminimyoclonus of fingers arose. Pulse rate at rest was 120 bpm.

Electrophysiological examination revealed sensorimotor peripheral neuropathy with combined axonal and demyelinating characteristics and normal central motor conduction times to arms and legs. CSF findings included elevated lactate (5.0 mmol/l; reference < 2.3) and protein levels (50 mg/dl; reference < 45) but no nucleated cells and a normal glucose/serum quotient of 0.7.

The multisystemic involvement suggested mitochondrial cytopathy and led to genetic diagnostics. Sequencing of the mitochondrial DNA and exome sequencing with focused analysis of genes for nuclear-encoded mitochondrial proteins did not discover disease-explaining mutations but revealed four heterozygous variants of unknown significance in *ATP13A2, NDFUS2, GAMT,* and *SLC25A38* which were unlikely to explain the cause of disease.

## The unexpected twist

**Fig. 1:**
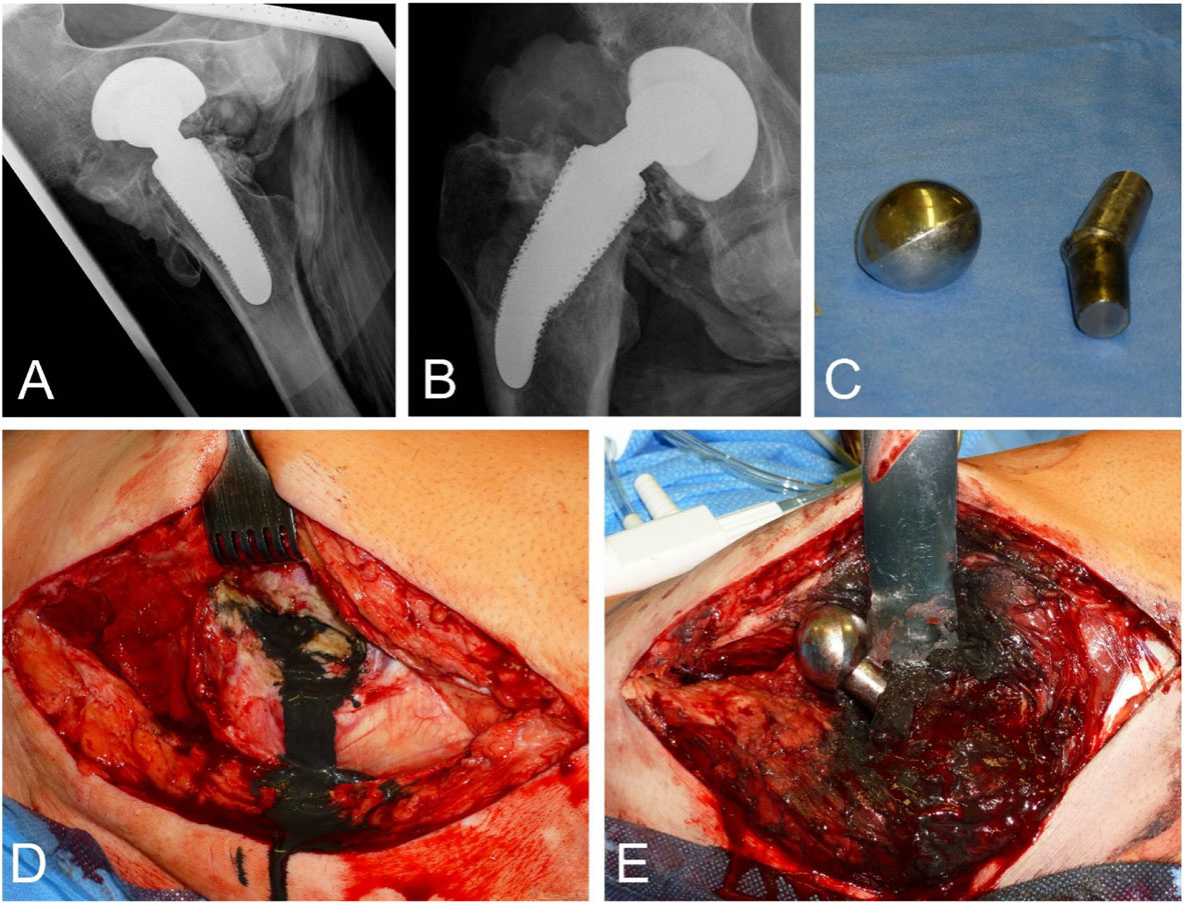
X-ray of the right hip joint in two planes: **A**) mediolateral projection and **B**) anterior-posterior projection show a decentered prosthesis head as evidence of inlay wear but no evidence of periprosthetic fracture or material loosening. Pronounced, periarticular heterotopic ossification (**A**) with bridging of the joint space (grade 4 according to Brooker). Flaw changes in the density of the peri-prosthetic region hint toward heavy metal abrasion. Streaky calcifications consistent with myositis ossificans (especially seen in **A**) in projection to the adductors and rotators indicate local inflammation probably related to metal abrasion. **C** Explanted femoral head and neck. Note the massive wear of the head, which was round at the time of implantation. **D** Leakage of black synovial fluid after removal of the hip joint capsule. **E** Massive metallosis and synovialitis after luxation of the hip joint. Abrasion of the femoral head of the prosthesis

The case remained unsolved for about 6 months when the patient returned with increasing hip pain to the department of orthopedics. X-ray of the hip is shown in Fig. 1. Ultrasound of the hip demonstrated prominent joint effusion and its puncture obtained a black sterile fluid. Surgical intervention for exploration and exchange of the defective implant revealed substantial shrinkage of the nickel-chromium-cobalt alloy prosthesis head due to abrasion. Screening for heavy metals disclosed massively increased chromium (25.0 μg/l - reference < 1.0) and cobalt levels (85.0 μg/l – reference < 7.0) in serum, which decreased in follow-up investigations after surgery (at 2 months: cobalt 75 μg/l, chromium 15.2 μg/l and at 6 months cobalt 36 μg/l, chromium 13.3 μg/l). No treatment was initiated by the surgeons, especially no cobalt lowering medication. Hip pain, as well as multisystemic impairment, improved significantly. One year after surgery, diabetes was well controlled without insulin (HbA1c 5.6%), gait unsteadiness ameliorated with fewer near falls, and vibration sense improved (5/8 ankles). Muscular wasting diminished, hair growth increased, and the patient gained 15 kg of weight. Visual acuity improved to ~ 30%. Pulse rate and blood pressure normalized even with reduced doses of a beta-blocker and ACE inhibitor. Hearing impairment remained unchanged but did not worsen further after surgery.

## Discussion

Iatrogenic heavy metal intoxication due to prosthetic abrasion is rare but can mimic multisystemic degenerative diseases, especially mitochondriopathies. To our knowledge, one case with similar symptoms has been reported previously [2] even though the symptomatic might be quite variable as reviewed by Gessner and colleagues [3]. In the series of 25 cases, symptoms were diverse and included beside hip (84%) the involvement of further systems like cardiovascular (60%), audiovestibular (52%), peripheral motor-sensory (48%), or the thyroid (48%)as most frequent affection. Also, psychological functioning (32%), visual impairment (32%), and hematological, oncological, or immune affection (20%) occurred. In this review [3], the mean cobalt levels were three times as high as in our patient and correlated well with symptom severity (r = 0.81, *p* > 0.001), but symptoms occurred with cobalt levels as low as 20 μg/l. Cobalt is cytotoxic to neural cells, which explains the peripheral neuropathy, including damage to our patient’s optic and acoustic nerves [4]. Cobalt ions destroy axonal mitochondria leading to axonal degeneration, including the optic nerve in rats [5]. Chromium has also been attributed to neurotoxicity [6], especially in cell- or animal models (fruit fly [7], zebrafish [8], and rat [9]), but in humans, symptoms are rather limited to hemotoxic and carcinogenic effects.

Prosthetic abrasion can be caused by ceramic shrapnel or splitter when failed ceramic prostheses are replaced by metal-on-metal prostheses. In non-failed metal-on-metal prostheses, increased cobalt levels (> 7 μg/l) have been measured in two thirds of the recruited sample of (*n* = 98) by Lodge and collegues [10]. In cases with cobalt levels > 7 μg/l subclinical cardiac abnormalities have been described [10]; we are not aware if studies focusing on neurological symptoms or subclinical neurological findings. In our case, no cobalt lowering therapy was initiated. There are some management suggestions with currently limited evidence as nicely discussed by Devlin and colleagues [11] of previously published reports of single cases, including EDTA treatment and 2,3-dimercaptopropane1-sulfonate (unithiol). Both cases had cobalt levels of > 500 μg/l, almost eight times the levels of our patient.

Since hip replacement is common and mitochondriopathies rare, it seems advisable to consider testing for heavy metal toxicity in the differential diagnostics of multisystemic disorders mimicking mitochondrial diseases.
